# Practice, perceived barriers and motivating factors to medical-incident reporting: a cross-section survey of health care providers at Mbarara regional referral hospital, southwestern Uganda

**DOI:** 10.1186/s12913-020-05155-z

**Published:** 2020-04-03

**Authors:** Turyahabwe Naome, Mwesigwa James, Atuhairwe Christine, Taremwa Ivan Mugisha

**Affiliations:** Clarke International University, P.O Box 7782, Kampala, Uganda

**Keywords:** Medical incident reporting, Practices, Motivating factors, Perceived barriers, Patient safety, Qualitative

## Abstract

**Background:**

Medical-incident reporting (MIR) ensures patient safety and delivery of quality of care by minimizing unintentional harm among health care providers. We explored medical-incident reporting practices, perceived barriers and motivating factors among health care providers at Mbarara Regional Referral Hospital (MRRH).

**Methods:**

We conducted a cross-sectional descriptive study on 158 health provider at Mbarara Regional Referral Hospital (MRRH), Western Uganda. Data was gathered using a structured questionnaire and analyzed with SPSS. The chi-square was used to determine factors associated with MIR at MRRH.

**Results:**

The results showed that there was no formal incident reporting structure. However the medical-incidences identified were: medication errors (89.9%), diagnostic errors (71.5%), surgical errors (52.5%) and preventive error (47.7%). The motivating factors of MIR were: establishment of a good communication system, instituting corrective action on the reported incidents and reinforcing health workers knowledge on MIR (*p*-value 0.004); presence of effective organizational systems like: written guidelines, practices of open door policy, no blame approach, and team work were significantly associated with MIR (p-value 0.000). On the other hand, perceived barriers to MIR were: lack of knowledge on incidents and their reporting, non-existence of an incident reporting team and fear of being punished (p- value 0.669).

**Conclusion:**

Medical Incident Reporting at MRRH was sub-optimal. Therefore setting up an incident management team and conducting routine training MIR among health care workers will increase patient safety.

## Background

Patient safety, that circumvents omission or commission resulting in unintended harm to the patient [1, 2] is fundamental principle to health care provision [2]. It is a core health concern of the *Primum non nocere* (‘first do no harm’) principle [1], and it is a universal foci of the public, healthcare providers (HCPs), and healthcare institutions [1, 2]. Patients’ safety is key to prevent the risk of medical incidences, defined as a deviation from usual medical care that may aggravate injury to the patient, increase disability, and pose life-threatening adverse events [2]. Medical incidents occurs whenever a healthcare provider fails to comply with medical guidelines within any of the healthcare systems, and may involve medicines, surgery, laboratory reports or equipment [3]. Adverse events are defined as an occurrence of unintended injury due to healthcare processes rather than the patient’s disease that may lead to permanent injury, prolonged hospitalization, and death [2, 3]. To achieve patients’ safety, the principles of Human Factors which involves the implementation of proactive medical incidents management systems that brings forth the occurrences that may threaten patient safety, utilize the system to enable understanding and learning from the incidences, to match these to routine medical practice is highly important [2–4]. While a substantial achievement of patient safety has been realized, there remains unacceptably high medical incidences, particularly in low- and middle income countries as care is often delivered in a pressurized and fast moving environment involving vast array of technology, and various individual decisions and judgments by HCPs [3–6]. According to World Health Organization (WHO), patient harm is among the high causes of the global disease burden, and is comparable to diseases such as tuberculosis and malaria [5]. Of the estimated 421 million global annual hospitalizations, about 42.7 million experience medical-incidences related to diagnostic, documentation, medication, prescribing, dispensing, drug administration, surgical, procedural, and decision-making [3, 6].

To address this, a multipronged approach that utilizes medical incident reporting (MIR) is key [5]. This is premised on the fact that MIR within healthcare eases identification and documentation of medical non-conformances that enables management to address as they arise, and information from reports enables causes to be identified and corrections made to prevent recurrence of similar incidents [4, 5]. Also, MIR seeks to identify system failures and HCPs need for training to institute corrective measures in the organization, and ensure patient safety is observed [5, 6]. HCPs practice of medical incidences and their reporting are essential factors to understand in order to perceive the medical incidences as an educational exercise and quality improvement tool in the provision of patient safety [7–9]. Based on research evidence, there is evidence to suggest that MIR is underutilized yet it would serve enormous benefits to healthcare provision [3, 6, 8]. Too often, neither HCPs nor organizations advise one other whenever an incident occurs, nor do they share what they have learned when an investigation has been carried out yet this would improve on the trust in the healthcare system [6, 7]. In Uganda, a study by conducted within two hospitals of Kabale Regional Referral Hospital and Itojo General Hospital showed that medical-incidences related to adverse drug reactions (ADRs) were under-reported, yet these were important contributors to patients’ morbidity and hospitalization [10]. Also, the fact that MIR has not been integrated into the training curriculum of HCPs, most medical-incidences are noticed and corrected at ones discretion and are often referred to as nonevents [3–5]. In effort to overcome the *status quo*, there are established phenomenon as reported by numerous researches to foster MIR and patient care. These include: a supportive environment, a culture of no blame and shame, professional collegiality and self-regulation [10–13]. Also, more evidence suggests that whereas aircraft crews were motivated to report incidences as they would be rewarded [14–16], health care providers were discouraged from reporting due to the doubt from organization’s use of data reported such as these would attract penalties when used as evidence for legal proceedings, negative organizational response (for example blaming, disciplinary actions), moral conscience of previous mistakes, providers’ emotional responses to errors such as feeling worried, guilty, and depression following serious errors [16–19]. The negative attributes associated with MIR coupled with the dynamic operation of medical care may favor non-disclosure of medical-incidences [16]. To avert this, motivating factors towards MIR are key, and studies have indicated that providing a conducive environment (through ensuring confidentiality and provision of appropriate feedback) as well as incenting the HCPs would alleviate the practice of MIR [11, 12, 20, 21]. To expand the knowledge of MIR, we explored medical-incident reporting practices, perceived barriers and motivating factors among HCPs at Mbarara Regional Referral Hospital (MRRH).

## Methods

### Operational definitions

#### Adverse event

Is an unintended injury that results in temporary or permanent disability, death or prolonged hospital stay, and is caused by healthcare management rather than by the patient’s underlying disease process.

#### Hazard

Any threat to safety for example unsafe practices, conduct, equipment, labels and names.

#### Incident (or adverse incident)

Any deviation from usual medical care that causes an injury to the patient or poses a risk of harm. They include errors, preventable adverse events and hazards [3].

#### Medical error

Failure of a planned action to be completed as intended (error of execution) or the use of a wrong plan to achieve an aim (error of planning). Errors can include problems in practice, products, procedures and systems [3].

#### Near miss

Serious error or mishap that has the potential to cause an adverse event but fails to do so because of chance or because it is intercepted [6].

#### Safety

Is a process or the act of professionalism to totally overhaul omission or commission that may result in hazardous healthcare conditions and/or unintended harm to the patient [2].

### Study design and reporting

We used a cross sectional study to determine the practice, perceived barriers and motivating factors to medical-incident reporting at MRRH in Southwestern Uganda. This study design was selected to give a snap shot of MIR among HCPs at a tertiary health facility.

### Study setting, population, sampling and sample size

This study was conducted at Mbarara Regional Referral Hospital (MRRH), one of the 13 Regional Referral Hospitals in Uganda that receives patients from within and other neighboring countries of Rwanda, Tanzania, Burundi and Democratic Republic of Congo. MRRH was purposively selected because it is the biggest hospital in the southwestern region offering comprehensive subspecialties (clinical and diagnostic services) and cares to an estimated 4 million people. In this study health workers that include: doctors, clinical officers, nurses, dispensers, laboratory personnel, pharmacists, theatre assistants, and anesthetists respectively were interviewed. Only 158 of 197 (80.2%) health care workers consented to take part in the study. The study population comprised HCPs at MRRH who had served for at least a year. Those who at the time of the study were away for leave were excluded. Census sampling was used in which all the HCPs were considered.

### Data instrument and collection

We gathered data using a self-administered questionnaire (Additional file), and an additional note was attached with each questionnaire which defined the aim of the research. The questionnaire comprised closed ended questions measured on a four-level Likert scale from 1 to 4 (Strongly agree, Agree, Disagree, and Strongly disagree, respectively). The questionnaire was developed from existing literature on earlier studies [8, 10–15]. It was structured into: Medication, surgical, and preventive incidences/errors ever witnessed at MRRH; individual practice of incidence/error reporting; motivating factors; and perceived barriers towards MIR. Two members of the research team assisted by two senior HCPs who had more than 10 years of experience in patient safety research assessed the questionnaire for face and content validity. Adjustments were made based on the need until such a point when it was deemed satisfactory, that it was even further pretested on thirty HCPs prior to administration to the study participants. The research team liaised with the staff on duty based on the existing monthly rosta, and a convenient time to consent and administer a questionnaire was chosen based on the HCPs availability and work schedule. The research team utilized the existing duty rooms for seeking participant’s consent during which the purpose of the study was explained to participants, and thereafter, a questionnaire was given out to read and fill in as a member of the research team was nearby in case one needed clarification for such a question(s) found difficult to understand. These were rephrased for a clear understanding by the interviewee. For cases that had reportedly witnessed a medical incident, the questionnaire included asking the participants whether or not they reported the medical incident.

### Study variables, population, recruitment criteria and sampling techniques

We extracted data on the types of incidents, motivating factors, and perceived barriers to MIR respectively. The dependent variable was medical incident reporting (MIR), and was measured as a binary outcome (Yes or No). Compliance to MIR was assessed based on the following questions: “For any of the errors/mistake above, what action was taken?” With answers (a) Reported and (b) Not reported. A health worker who was directly or indirectly affected by an incident promptly reported it so that appropriate corrective action was taken; this was considered to have reported the medical incidence. And “If you reported, when did you report the incident?” with answers (a) Immediately (within 24 h) and (b) Later (more than 24 h or never). Conversely, any health worker that observed or was injured or witnessed an injury to a patient or staff but did not report the incident were considered non-compliant. These were finally categorized as “YES” and “NO” (by merging the responses from the 2 questions as self-reported responses). The study population comprised HCPs at MRRH who had served for at least a year. Census sampling was used in which all the HCPs were considered. Selection was aimed at ensuring that HCPs from all departments were considered. Specifically, the questionnaires were distributed to cover at least 50% of the HCPs in a department to provide a representative sample. These were based on the different specialties, gender, and seniority. In circumstances where the first contact declined to participate in the study, someone else in the same department with a similar characteristic was contacted.

### Quality control

The questionnaire was pre-tested among thirty consented HCPs within two health centres (HCs) in Mbarara Municipality, namely: Mbarara Municipal Council HC IV, and Kakoba Health Centre III (HC III). Quality of data was guaranteed through self-administered questionnaires to avoid any bias, and the research assistants were available and within close range to the respondents, in case the latter needed clarity. The research assistants checked and ensured the completeness of the questionnaire.

### Data analysis

The data gathered was entered into SPSS (statistical software for social scientist), cleaned, then analyzed for univariate, and bivariate at 5% significance level. At univariate analysis, descriptive statistics in the form of frequencies and percentages were used for categorical variables. To establish which factors were highly correlated to MIR, the principle component analysis (PCA) was used a data reduction tool. A variable that had a component loading of greater than 0.60 was considered to correlate to MIR at MRRH. While the Bartlett’s test (approximation of the Chi-square test) was used to test whether the variances in the sample were equal across the groups. Only associations with probability value (*P*-value) less than 5% were considered to be statistically significant in determining MIR at MRRH.

### Ethical considerations

As this study did not involve human sampling or clinical procedures/intervention otherwise considered by Uganda National Council for Science and Technology to interfere with humans [22], it received an exemption of ethical clearance from Clarke International University (UG-REC-015). However, administration approval was obtained and this was presented to the Director MRRH, who then authorized the conduct of this research. In this study, HCPs consent was obtained from all participants. Their responses were treated with utmost confidentiality and not linked to participant’s identity.

## Results

Among the 197 healthcare providers, 158 were interviewed giving 80.2% coverage. Of these, majority were nurses (*N* = 105, 66.5%), while other professions were: laboratory personnel 7 (4.4%), midwives 12 (7.6%), doctors 14 (8.9%), clinical officer 12 (7.6%), pharmacist 2 (1.3%), anesthetist 3 (1.9%), dispenser 1 (0.6%) and theatre assistant 2(1.3%). Numerous medical incidents were reported: a) Medication errors which were mainly in form of drugs administered at wrong time (118/158, 74.7%); b) Diagnostic errors, mostly due to delayed diagnosis (134/158, 84.8%), and failure to use laboratory results appropriately (109/158, 69.0%); c) Preventive errors, majorly as a result of failure to provide prophylactic treatment (110/158, 69.6%); d) Surgical errors, mainly as a result of pre-operative management (50/158, 31.6%), and omitted pre-operative investigations (45/158, 28.5%); and d) Other uncategorized incidences, that were predominantly manifested as: drug reactions (45/158, 28.5%), and equipment failure (30/158, 19.0%). The pattern of various incidences are summarized in Table 1. Table 2 shows the individual motivating factors and medical incident reporting. Majority of the health workers (92/158, 58.2%) agreed that Supportive environment of no blame and shame encourage adherence to incident reporting. More than half (94/158, 59.5%) of the health workers agreed that having knowledge on how, what and whom to report incidents influences adherence, about 67/158(42.4%) of them agreed to giving rewards encouraged incident reporting, 88/158(55.7%) of the health workers in the study agreed that providing corrective actions about incidents report increased reporting; 85/158 (55.7%) said that training health provider to identify medical incidents motivated incident reporting and only 65/158 (41.1%) reported that establishing communication channels with emphasis on feedback promoted medical incidents. The Bartlett’s test showed a positive association between individual motivation factors and medical incident reporting at Mbarara Regional Referral Hospital (p-0.004).

**Table 1:** Showing the patterns of medical incidents at MRRH

Profession	Frequency (n)	Percentage (%)
Nurses	105	66.4
Laboratory technician	7	4.4
Midwives	12	7.6
Doctors	14	8.9
Clinical officer	12	7.6
Pharmacist	2	1.3
Anesthetist	3	1.9
Theatre assistant	2	1.3
Dispenser	1	0.6
Total	158	100.0
Category of incidence *(multiple responses question)*	**Frequency (n/158)**	**Percentage (%)**^**a**^
Medication errors		
Wrong dose	80/158	50.6
Poly pharmacy	100/158	63.3
Drug administered at the wrong time	118/158	74.7
Wrong route	60/158	38
Diagnostic errors		
Wrong diagnosis	26/158	16.5
Delayed diagnosis	134/158	84.8
Inappropriate investigation	101/158	63.9
Failure to use results	109/158	69.0
Preventive errors		
Failure to provide prophylactic treatment	110/158	69.6
Others	15/158	9.5
Surgical errors		
Pre-operative management	50/158	31.6
Omitted pre-operative investigation	45/158	28.5
Forgotten materials in the body	10/158	6.3
Omitting post-operative notes	35/158	22.2
Others	5/158	3.2
Otherwise uncategorized incidences		
Drug reactions	45/158	28.5
Equipment failure	30/158	19.0
Near misses	6/158	3.8

^a^Percentages are rounded off to one decimal place

**Table 2:** Individual motivating factors that influenced incident reporting (*n* = 158)

Statement	SA	A	D	SD
Supportive environment of no blame and shame encourage incident reporting	64 (40.5)	92 (58.2)	0 (0.0)	2 (1.3)
Knowledge on how, what and whom to report incidents influences	53 (33.5)	94 (59.5)	10 (6.3)	1 (0.6)
Rewarding of those who report incidents encourages reporting	43 (27.2)	67 (42.4)	35 (22.2)	13 (8.2)
Providing corrective actions about the reported incidents	48 (30.4)	88 (55.7)	14 (8.9)	8 (5.1)
Training on incidents motivates incident reporting.	41 (25.9)	85 (53.8)	22 (13.9)	10 (6.3)
Proper communication with emphasis on feedback promotes incident reporting	31 (19.6)	65 (41.1)	45 (28.5)	17 (10.8)

*SA* Strongly Agree, *A* Agree, *D* Disagree, *SD* Strongly Disagree

Bartlett’s test approx. Chi-square = 33.616; level of significance (probability value) = 0.004

In Table 3; the results show that the existence of reporting system greatly influences medical incident reporting (90/158, 57.0%). The health care workers also strongly agreed that the presence of written guidelines promoted incident reporting in an organization (95/158, 60.1%). Majority (101/158, 63.9%) of the health workers strongly agreed that the practice of teamwork in a hospital promoted incident reporting. In addition the accessibility to ward in-charges and administrators (open door policy) strongly influenced incident reporting (115/158, 72.8%). Finally, 110/158(69.6%) of the health workers strongly agreed that the practice of no blame approach to those who report incidents influenced incident reporting.

**Table 3:** Organizational motivating factors that influenced incident reporting

Statement	SA	A	D	SD
The existence of a reporting system influences incident reporting	90 (57.0)	40 (25.3)	22 (13.9)	6 (3.8)
Presence of written guidelines promotes incident reporting in an organization	95 (60.1)	46 (29.1)	13 (8.2)	4 (2.5)
Practice of teamwork in a hospital promotes incident reporting	101 (63.9)	32 (20.3)	17 (10.8)	8 (5.1)
Accessibility to ward in-charges and administrators (open door policy) influences incident reporting	115 (72.8)	34 (21.6)	5 (3.2)	4 (2.5)
Practice of no blame approach to those who report incidents influences incident reporting	110 (69.6)	28 (17.7)	11 (7.0)	9 (5.7)

*SA* Strongly Agree, *A* Agree, *D* Disagree, *SD* Strongly Disagree

Bartlett’s test approx. Chi-square = 34.843; level of significance (probability value) = 0.000

With regard to perceived barriers to medical incident reporting, majority (83/158, 52.5%) strongly agreed that lack of knowledge about incidents was a barrier to incident reporting. The absence of an incident management team in the hospital has greatly affected medical incident reporting (85/158, 53.8%). The health workers also reported that the failure of the administration to keep confidentiality of those that report incidents was a barrier to prompt incident reporting (93/158, 58.9%). Lack of support from the management of the hospital was a barrier to health workers reporting medical incidents (68/158, 43.0%). Isolation by fellow staff after one has reported an incident was another key barrier to medical incident reporting at MRRH (64/158, 40.5%). Regarding administrative punishment as a common barrier to incident reporting, 103/158(65.2%) of them agreed it was a common barrier at MRRH. However, no significant association was observed between the perceived barriers and medical incident report at Mbarara regional Referral Hospital (p-0.669). The decision to report was based on varied individual and organizational motivating factors as given in Tables 2 and 3, respectively. Further, there were numerous perceived barriers that deterred MIR, as presented in Table 4. The principal component analysis (PCA) was used as a data reduction tool to reduce the observable correlated variables to a smaller set of independent component factors. Only factors with a component loading greater than 0.6 were considered to be highly corrected to MIR and the results are summarized in Table 5. These variables include: having a good communication system (.749), presence of a corrective action in the system (0.614), knowledge on incidents (0.696), presence of written guideline (0.715), presence of an open door policy (0.691), practice of teamwork (0.605), lack of knowledge of the importance of medical incident reporting (0.655), absence of an incident report team (0.686) and fear of being punished by the administrator (0.630). The perceived barriers to incident reporting indicated that lack of knowledge on incidents and their reporting, absence of an incident reporting team and fear of being punished by the administrators were hindrances at MRRH, as shown in Table 6.

**Table 4:** Perceived barriers to incident reporting

Statement	SA	A	D	SD
Lack of knowledge about incidents is a barrier to incident reporting	83 (52.5)	45 (28.4)	7 (4.4)	23 (14.6)
Absence of an incident management team in the hospital	46 (29.1)	85 (53.8)	22 (13.9)	5 (3.2)
Failure of the administration to keep confidentiality of those that report incidents	35 (22.2)	93 (58.9)	26 (16.5)	4 (2.5)
Lack of support from the management of the hospital to health workers about incident reporting	54 (34.2)	68 (43.0)	27 (17.1)	9 (5.7)
Fear of being punished by the administration	34 (21.5)	103 (65.2)	15 (9.5)	6 (3.8)
Isolation by fellow staff after one has reported an incident	57 (36.1)	64 (40.5)	25 (15.8)	12 (7.6)

*SA* Strongly Agree, *A* Agree, *D* Disagree, *SD* Strongly Disagree

Bartlett’s test approx. Chi-square = 12.126; level of significance (probability value) = 0.669

**Table 5:** Showing motivational factors

	Component		
Individual motivating factor (Strongly agree, agree, disagree, strongly disagree)	1	2	3
Rewards and incentives to health workers	.297	.790	.235
Good communication system	.749^a^	−.135	−.393
Corrective action in the system	.614^a^	.181	.354
Training of health workers on incidents	.397	.284	.508
Support from administration	−.131	.656	−.658
Knowledge on incidents	.696^a^	−.071	.222
**Organizational factors**			
Presence of a reporting system	.358	.364	−.640
Presence of written guideline	.715^a^	.088	−.413
Presence of an open door policy	.691^a^	−.622	.077
Presence of a no blame approach	.286	.793^a^	.388
Practice of team work	.605^a^	.017	.596
Extraction Method: Principal Component Analysis			

**NB:**

1. Components 1, 2 and 3 represent how independent variables correlate with MIR

2. ^a^ Interpreted as correlations and only correlations above > 0.60 where considered (+ 1 to −1) to be moderately or fairly strongly correlated to MIR in Mbarara Regional Referral Hospital (MRRH)

**Table 6:** Perceived barriers to incident reporting

	Component		
Barrier to incident reporting (Strongly agree, agree, disagree, strongly disagree)	1	2	3
Lack of knowledge on incident reporting	.655^a^	−.290	−.117
Absence of an incident report team	.031	.686^a^	.545
Lack of confidentiality	.041	.591	−.337
Lack of management support	.239	−.186	.798
Fear of being punished by the administrator	.630^a^	.467	−.142
Lack of supportive staff	−.705	.150	.040
Extraction Method: Principal Component Analysis			

**NB:**

1. Components 1, 2 and 3 represent how independent variables correlate with MIR

2. ^a^Interpreted as correlations and only correlations above > 0.60 where considered (+ 1 to −1) to be moderately or fairly strongly correlated to MIR in Mbarara Regional Referral Hospital (MRRH)

## Discussion

From this study, medication errors were the major medical incidents reported at 89.9%. These were in form of wrong dose, polypharmacy and wrong route (50.6, 63.3 and 38.8%, respectively). These results are in agreement with the previous studies [23–25]. Among the diagnostic errors, delayed diagnosis was high (84.8%), wrong diagnosis, inappropriate investigation and failure to use results as recorded (16.5, 63.9, and 69%, respectively) were listed. Also, failure to provide prophylactic treatment was observed among 69.6% of the healthcare providers; these results are in agreement with previous studies [10, 26–28]. This finding is consistent with a report in which medical errors where reportedly the main contributory factors towards the patients harm [29]. In this, it was elucidated that even with appropriate medical care, patients are harmed during the process of receiving care [29]. This phenomenon is critical, and requires careful consideration to ensure patients safety. Majority of the healthcare workers (98.7%) agreed that a strongly supportive environment of no blame and shame would motivate them to report incidents, similar to a report by Tumwikirize et al. [10]. This implies that healthcare workers in away are concerned of the harm afflicted to patients under their care as a consequence of medical error. This phenomenon, earlier described as ‘second victim’ was shown to associate with emotional consequences to the healthcare worker [29]. Also, the perceived lack of value in incident reporting as when an event is reported, it feels like the person is written up, not the problem, as earlier highlighted [30, 31]. Knowledge on what, how and who to report to the incidents that do occur was found key as this was supported by 93% of the respondents who agreed. This is quite obvious that one may never report anything about what he/she does not understand as well as whom, and how to report it. These findings correlates well with a previous report [13, 16, 26–28]. Guided by the established system analysis of the clinical incidents [30], a comphrensive approach to analyze and investigate medical incident, going beyond the routine identification of the fault and apportioning blame is critical [30]. In this context, a closer analysis is ideal to explore the series of events that determine the incident occurrence; and significantly, use of systematic and structured approach to investigate and facilitate corrective actions [30]. This is attainable through such approaches that promote open discussion and subvert apportioning blame [30].

Additionally, incenting those who report incidents, providing corrective action of the reported incidents, training of health workers about incidents and proper communication about the reported incidents with emphasis on prompt feedback were in agreement with previous studies [32, 33]. Bivariate analysis indicated that three factors that strongly motivated health workers to report medical incidents. These include presence of a good communication system, ensuring corrective action on the reported incidents and ample knowledge about medical incidents and their reporting similar to previous reports [6, 12–14, 27]. The study identified organizational factors like presence of a reporting system, presence of written guidelines on MIR, practice of team work, accessibility of ward in charges (open door policy) and practice of no blame approach to MIR [18, 19, 21, 26]. A reporting system helps to guide healthcare workers on how and where to report incidents; and it also helps to analyze, investigate, and disseminate information about reported incidents and hence their recurrence. Also, open door policy where in-charges are accessible to health care workers is critical as such kind of administration greatly influences incident reporting since health workers don’t have any fear of administrative punishment. These finding are in agreement with earlier reports [3, 5, 6, 24–27].

The barriers to MIR indicated that majority (80.9%) of the health workers indicated lack of knowledge. To support this, on job trainings and inclusion of MIR in the curriculum of medical training could enhance the understanding of MIR, as already recommended by WHO [3]. Another barrier to MIR was lack of an incident management team. This in a way may deny a chance of investigating, analyzing and disseminating information about incidents. This correlates with findings from previous studies [8, 10, 14]. Failure of the administration to keep confidentiality of those who report incidents, fear of administrative punishment, isolation by fellow health workers and lack of administrative support were other hindrances. This implies that a health worker would never report an incident when he/she is not sure of its consequences which can include a legal punishment, being terminated from the job and putting a copy of a letter in one’s file. These are in agreement with previous reports [12, 14, 21, 28, 31, 34]. Although this study was conducted in a single regional referral hospital, its results could to some degree be generalizable to the other settings considering the fact that the healthcare system and curriculum of healthcare professionals is similar across the country. The findings of this study ought to be interpreted in light of the following: a) the study relied on respondent self-reporting, thus there may have been some source of information bias due to fear of being reprimanded; b) There may have been a possibility of self-report bias due to self-administered questionnaire; c) we did not conduct qualitative interviews to triangulate these research findings.

## Conclusion

The study found out that the commonly witnessed medical incidents were medication, diagnostic, surgical and preventable errors. The factors that would strongly motivate health workers to report incidents were: good communication system, ensuring corrective action on the reported incidents and boosting the knowledge of healthcare providers in regard to medical incidents and their reporting. Also, organizational factors that would strongly influence MIR were: written guidelines, practice of open door policy, no blame approach and ensuring team work. The strongest barriers to incident reporting were: lack of knowledge, absence of an incident reporting team and fear of being punished by the administrators. Based on these findings, there is urgent need to refocus and design strategies to promote MIR practice by harnessing in-service training of health workers. Also, there is need to redesign the training contents to include patient safety in the curriculum. It is vital that an incident reporting team is established to ensure that healthcare providers and the administration are trained about incidents and MIR.

## Supplementary information

**Additional file 1.** Questionnaire.

## Data Availability

We did not obtain consent to share data obtained, however the datasets used and/or analyzed during the current study is available from the corresponding author on reasonable request.

## Abbreviations

ADRs: Adverse Drug Reactions
HC: Health Centre
HCPs: Healthcare providers
MIR: Medical Incidence Reporting
MRRH: Mbarara Regional Referral Hospital
PCA: Principle Component Analysis
SA: Strongly Agree
A: Agree
D: Disagree
SD: Strongly Disagree
WHO: World Health Organization
