# Supplementary material for: Practice, perceived barriers and motivating factors to medical-incident reporting: a cross-section survey of health care providers at Mbarara regional referral hospital, southwestern Uganda
Source: BMC Health Serv Res. 2020 Apr 3;20:276. doi: 10.1186/s12913-020-05155-z (PMC7118859; doi:10.1186/s12913-020-05155-z)
Supplement: Supplementary file 1 — Additional file 1. Questionnaire. [file 12913_2020_5155_MOESM1_ESM.docx]

## **Questionnaire**

**SECTION A**

Qn1. Which of the following medical errors/mistakes below have you ever witnessed in this hospital? (Tick appropriately)

**a) Medication errors:**

i. Wrong dose ii. Polypharmacy iii. Wrong time of drug administration iv. Wrong route

v. Wrong patient vi. Others (specify)…………………………………………

**b) Surgical errors**

i) Pre-operative management

- No consent

- No pre-medication (including noting time of administration)

- No starvation

ii) Omitted pre-operative laboratory investigation. iii) Wrong site surgery

iv) Forgotten materials in patients’ body

v) Omitting post-operative notes/ instructions by the surgeon

vi) Others (specify)……………………………………….

**c) Preventive errors**

i) Failure to provide prophylactic treatment ii) Others (specify)………………

d) **Diagnostic errors**

i) Wrong diagnosis ii) Omitted diagnosis iii) Delayed diagnosis

iv) Inappropriate investigation v) Failure to use results vi) Others specify…………

**Practice of error reporting**

Qn.2. For any of the errors/mistake above, what action was taken?

a) Reported b) Not reported

i). If you reported, when did you report the incident?

1. Immediately (within 24 hours) b) Later (more than 24 hours)

**SECTION B**

1) Rewards and incentives to health workers: Being rewarded and given incentives after reporting a medical error would make me to always report incidents. a) Strongly agree b) agree c) disagree d) strongly disagree

2) Good communication system: Presence of a good communication system in the hospital about medical error reporting would encourage me to report such errors always.

1. Strongly agree b) agree c) disagree d) strongly disagree

3) Corrective action in the system: If an error is reported and a corrective action is taken about it, I would always be encouraged to report errors.

1. Strongly agree b) agree c) disagree d) strongly disagree

4) Training of health workers on incidents: On job training about medical errors, medical error reporting is important in ensuring that I report errors. a) Strongly agree b) agree c) disagree d) strongly disagree

5) Support from administration: A supportive administration that walks the talk of medical error reporting without blaming those who make and report errors would encourage me to report errors.

1. Strongly agree b) agree c) disagree d) strongly disagree

6) Knowledge on incidents: Being knowledgeable about medical errors is a motivating factor to medical errors

1. Strongly agree b) agree c) disagree d) strongly disagree

**SECTION C**

1. Presence of a reporting system: Presence of written guidelines in a hospital would influence me to report errors.
2. Strongly agree b) agree c) disagree d) strongly disagree

2) Presence of written guideline: Presence of a clear medical error reporting system in the hospital would influence me to report medical errors. a) Strongly agree b) agree c) disagree d) strongly disagree

3) Presence of an open door policy: An approachable hospital leadership would indeed influence me to report errors.

a) Strongly agree b) agree c) disagree d) strongly disagree

4) Practice of team work: Practice of a team work spirit among health workers can influence health workers to report medical errors. a) Strongly agree b) agree c) disagree d) strongly disagree

5) Presence of a no blame approach: Practice of no blame approach by the hospital administration towards those who report medical errors can influence health workers to always report errors.

1. Strongly agree b) agree c) disagree d) strongly disagree

**SECTION D**

1. Lack of knowledge on incident reporting: Lack of knowledge about medical errors and medical error by health workers is a barrier to error reporting in this hospital.
2. Strongly agree b) agree c) disagree d) strongly disagree
3. Absence of an incident report team: Absence of a team in charge of medical error reporting is also a barrier to incident reporting in this hospital. a) Strongly agree b) agree c) disagree d) strongly disagree
4. Lack of management support: Lack of support from the management on medical error reporting is a barrier to error reporting in this hospital. a) Strongly agree b) agree c) disagree d) strongly disagree
5. Lack of confidentiality: Fear of being isolated by fellow health workers after reporting an incident is another barrier to medical error reporting in this hospital.
6. Strongly agree b) agree c) disagree d) strongly disagree
7. Lack of supportive staff: Fear of being blamed after reporting an incident is a barrier to incident reporting in this hospital. a) Strongly agree b) agree c) disagree d) strongly disagree
8. Fear of being punished by the administrator: Fear of being punished by administration as having committed an error and reporting it is also a barrier of error reporting in this hospital.
9. Strongly agree b) agree c) disagree d) strongly disagree
